# *Lycium barbarum* Polysaccharide and Chlorogenic Acid Ameliorate LPS‐Induced Acute Lung Injury via the NF‐κB Signaling Mediated Multi‐Targets

**DOI:** 10.1002/fsn3.71438

**Published:** 2026-01-07

**Authors:** Ziyue Wang, Jianxiong Hao, Junye Yin, Huan Rao, Xia Zhao, Lulu Yu, Dandan Zhao

**Affiliations:** ^1^ College of Food Science and Biology Hebei University of Science and Technology Shijiazhuang China; ^2^ Department of Psychiatry The First Hospital of Hebei Medical University Shijiazhuang China

**Keywords:** acute lung injury (ALI), chlorogenic acid (CA), *Lycium barbarum* polysaccharide (LBP), network pharmacology, NF‐κB signaling

## Abstract

Acute lung injury (ALI) is a life‐threatening condition characterized by acute inflammatory damage to the alveolar epithelium and capillary endothelium, triggered by various pathological factors. Our previous study demonstrated the anti‐inflammatory and anti‐oxidative effects of *Lycium barbarum* polysaccharide (LBP) and chlorogenic acid (CA) against lipopolysaccharides (LPS)‐induced inflammatory response of NR8383 cells, while the current study further investigated the alleviating effect of LBP and CA against ALI through in‐depth experiments. In vitro experiments identified potential targets (*CASP3*, *KDR*) and related pathways, while in mice with ALI induced by 5 mg/kg LPS, CA‐LBP (CA:LBP = 1:7, L/M/H dose: 25/50/100 mg/kg) was administered for 14 consecutive days, which reversed weight loss, alleviated lung injury, suppressed MPO activity, reduced TNF‐α, IL‐1β, IL‐6 and MDA levels, enhanced SOD and GSH‐Px activities, and mitigated NF‐κB activation significantly. Data of molecular docking further confirmed that the CA‐LBP complex can exert alleviating effects through suppressing the NF‐κB signaling pathway. Taken together, the present work sheds light on the multi‐target regulatory mechanism of CA‐LBP against ALI.

## Graphical abstract

*Lycium barbarum* polysaccharide (LBP) and chlorogenic acid (CA) ameliorate LPS‐induced acute lung injury (ALI) through a multi‐target regulatory mechanism, which involves suppression of the NF‐κB signaling pathway.

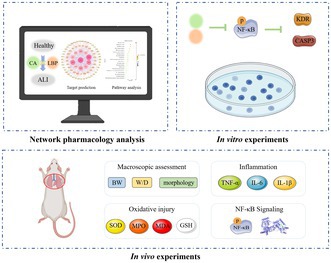

**Abbreviations**
ALI: acute lung injury
BP: biological processes
CA: chlorogenic acid
CASP3: caspase 3
CC: cellular components
EGFR: epidermal growth factor receptor
GSH‐Px: glutathione peroxidase
IL: interleukin
KDR: vascular endothelial growth factor receptor 2
LBP:
*Lycium barbarum* polysaccharide
LPS: lipopolysaccharide
MDA: malondialdehyde
MF: molecular functions
MMP2: matrix metalloproteinase 2
MPO: myeloperoxidase
NF‐κB: nuclear factor kappa‐B
SOD: superoxide dismutase
TNF‐α: tumor necrosis factor‐α

## 1 Introduction

Acute lung injury (ALI), a therapeutically challenging respiratory disorder featured by uncontrolled inflammatory cascades, oxidative stress, and alveolar‐capillary barrier disruption, remains a principal factor of fatal outcomes in patients under critical care (Cao et al. 2025; Leist et al. 2020; Li, Hou, et al. 2023). Despite advances in clinical management, such as protective ventilation and glucocorticoid therapy, existing treatments often fail to address the multifactorial pathogenesis of ALI and are associated with adverse effects, including immunosuppression and bone density loss (Ding et al. 2020; Liu et al. 2024). This underscores the urgent need for safer, multi‐target therapeutic strategies that synergistically modulate inflammation and oxidative stress.

Natural bioactive compounds, particularly polyphenols and polysaccharides, have emerged as promising candidates for managing complex diseases like ALI due to their multi‐pathway regulatory capabilities (Wu et al. 2023), making them promising in medicine and health products (Wei et al. 2025). Polyphenols, secondary metabolites with diverse phenolic structures, are major dietary phytochemicals. According to the chemical structure, they can be categorized into two main groups: flavonoids and non‐flavonoids (Farid et al. 2025; Li, Chen, et al. 2023). Chlorogenic acid (CA), a common phenolic acid in daily diets, has been identified in plants like honeysuckle, eucommia, coffee beans, and tea (Zeng et al. 2022). Polysaccharides like *Lycium barbarum* polysaccharides (LBP), water‐soluble polysaccharides and major active components of goji berries, consist of carbohydrate chains and proteins (Liu et al. 2022). Notably, both CA and LBP share a range of beneficial pharmacological properties, including potent antioxidant, anti‐inflammatory, and immunomodulatory effects. CA acts by scavenging free radicals and modulating signaling pathways such as nuclear factor kappa‐B (NF‐κB) and Nrf2 (Nguyen et al. 2024; Ye et al. 2025), while LBP has also demonstrated anti‐cancer and gut microbiota‐modulating activities (Kou et al. 2022; Liu et al. 2021). Given their shared anti‐inflammatory and antioxidant profiles, both CA (Zhang et al. 2010) and LBP (Ren et al. 2023) have exhibited remarkable potential in mitigating pulmonary inflammation and injury, which forms the foundation of this study. Furthermore, recent studies suggest that combining polyphenols and polysaccharides may amplify their bioactivity via structural interactions, such as hydrogen bonding between phenolic hydroxyl groups and polysaccharide functional groups, as well as multi‐target synergism (Xue et al. 2024).

Our previous study revealed that CA‐LBP complexes have shown enhanced antioxidant and anti‐inflammatory bioactivity compared to individual components, highlighting their potential for ameliorating oxidative stress and inflammation‐related disorders (Yin et al. 2024). These findings inspired our interest in investigating the potential of CA‐LBP complexes for protecting ALI. Network pharmacology, a branch of systems pharmacology, studies drug‐disease interactions via complex biological network analysis. Moreover, it performs the screening of effective ingredients, prediction of targets, building of multi‐dimensional networks, and examination of data for complex regulatory networks (Wu, Zhang, et al. 2022). Given the multi‐component nature of CA‐LBP and the complex pathogenesis of ALI, this method is uniquely suited to identify potential targets. Thus, it provides crucial insights for exploring the targets of CA‐LBP in ALI in this study. Meanwhile, the in‐depth investigation into the alleviating effects of CA‐LBP on ALI deserves further research.

However, current research on CA and LBP in ALI remains limited. Most studies focus on their individual effects, with insufficient exploration of their combined application and underlying synergistic mechanisms. Based on our prior findings, this study employed network pharmacology to explore CA and LBP targets in ALI, established the in vivo and in vitro models, and applied diverse molecular biology techniques to uncover the mechanism of CA‐LBP against ALI, seeking to offer novel therapeutic insights into ALI and expand the potential applications of CA and LBP.

## 2 Materials and Methods

### 2.1 Materials and Reagents

LBP extract and lipopolysaccharides (LPS) were gained through Solarbio Science and Technology (Beijing, China). CA was obtained through Aladdin Biochemical Technology (Shanghai, China). Specific primary antibodies of p‐p65, β‐actin, and AP‐labeled Goat Anti‐Rabbit IgG secondary antibodies were purchased through Bioworld Technology (Nanjing, China).

### 2.2 Target Gene Prediction

Three‐dimensional chemical structures of LBP and CA were obtained from the online PubChem database. The potential target identifications of LBP and CA were completed using the Swiss Target Prediction database (top 15 targets per compound, probability ≥ 0.5). The target identification of ALI disease was obtained through the DisGeNet (score ≥ 0.1) and GeneCards (relevance score ≥ 5) databases. The putative targets of LBP and CA were intersected with the targets related to ALI, and these overlapping targets were regarded as potential targets of LBP and CA acting on ALI disease.

### 2.3 Protein–Protein Interaction Network (PPIN) Analysis

To evaluate the potential physical and functional association between the potential targets of LBP and CA acting on ALI, PPIN functional enrichment analysis of the intersected targets was conducted with the STRING 11.5 database (interaction confidence score ≥ 0.7). Then, Cytoscape software 3.10.1 (Bethesda, USA) was utilized to calculate the degree values in the PPIN. Then, the “Cytohubba” was employed to screen out the top 10 core target points.

### 2.4 Gene Ontology (GO) and Kyoto Encyclopedia of Genes and Genomes (KEGG) Enrichment Analysis

To understand the potential biological functions of the target points of LBP and CA acting on ALI, GO and KEGG enrichment analysis were carried out with the Metascape database the default significance thresholds (minimum term size 3, *p* ≤ 0.01, FDR ≤ 0.05). The results were visualized with WeiShengXin (https://www.bioinformatics.com.cn/).

### 2.5 Cells and Treatment

Rat alveolar macrophages (NR8383) were supplied by the Pricella Biological Company (Wuhan, China). The procedure of cell culture was detailed in our previous research (Yin et al. 2024). Cells were used within 10 passages and seeded at a density of 1 × 10^6^ cells/mL. Briefly, at 37°C with 5% CO_2_, cells were cultured in Ham's F‐12k complete medium containing 20% FBS and 1% penicillin–streptomycin. Cells were grouped into the control, model (1 μg/mL LPS), CA‐LBP intervention (pretreated with 40 μg/mL CA, 280 μg/mL LBP, and 40 μg/mL CA + 280 μg/mL LBP for 24 h, and then stimulated with 1 μg/mL LPS for 24 h respectively) groups. The vehicle control was Ham's F‐12k complete medium without any treatment.

### 2.6 Animals and Treatment

Following a week of acclimation, SPF standard male Kunming (KM) mice aged 6–8 weeks were randomly assigned to 8 groups (*n* = 6): control group, Dex group (dexamethasone positive control); model group; CA group; LBP group; CA + LBP‐L/M/H groups.

From day 1 to 14, oral gavage was administered daily at a fixed time for two weeks prior to LPS‐induced modeling for the dose groups (CA (6.25 mg/kg), LBP (43.75 mg/kg), CA + LBP‐L/M/H (25/50/100 mg/kg, CA:LBP = 1:7)), while control, Dex and model groups were gavaged with saline. On day 15, the Dex group received dexamethasone (2 mg/kg) intraperitoneally injected. 1 h after administration, mice of the Dex, model, CA, LBP, and CA + LBP‐L/M/H groups were anesthetized with 10% chloral hydrate solution (0.045 mL/kg) and fixed. Tracheal intubation was performed using a venous indwelling needle, followed by intratracheal instillation of LPS (5 mg/kg). After 12 h of LPS treatment, once the modeling was confirmed to be successful, the mice were sacrificed and the organs were carefully isolated and stored.

### 2.7 Hematoxylin–Eosin (HE) Staining

Lung tissues were fixed in 4% formaldehyde for 48 h. After dehydration and clearing, embed the tissues with paraffin and cut the tissues into 5 μm sections. Followed by baking, sections were dewaxed, washed, stained, and sealed under the instruction of the HE staining kit (Solarbio Science & Technology Co. Ltd., Beijing, China). Observe the sections under the optical microscope.

### 2.8 Determination of Inflammatory Factors

The levels of tumor necrosis factor‐α (TNF‐α), interleukin‐1beta (IL‐1β), and interleukin‐6 (IL‐6) in mouse serum were measured using a commercial mouse enzyme‐linked immunosorbent assay (ELISA) kit. The methodology of the detection was in accordance with the instructions of the kit's manufacturer (Elabscience Biotechnology Co. Ltd., Wuhan, China).

### 2.9 Detection of Lung Biochemical Index

The measurement of myeloperoxidase (MPO), malondialdehyde (MDA), superoxide dismutase (SOD), and glutathione peroxidase (GSH‐Px) in mice lung tissues was operated in accordance with the instructions of the kit's manufacturer (Nanjing Jiancheng Bioengineering Institute, Nanjing, China).

### 2.10 Western Blot Analysis

After the extraction of total protein from cells and lung tissues, an equal mass of protein from each sample was loaded into the SDS‐PAGE gel. Subsequently, the samples were separated by gel electrophoresis and transferred onto the polyvinylidene fluoride (PVDF) membranes. Following the blocking, the membranes were incubated with specific primary antibodies and secondary antibodies in sequence (Table 1). Finally, we added the enhanced chemiluminescence (ECL) substrate (Beyotime Biotechnology, Shanghai, Beijing), captured the signal of the protein band in membranes, and analyzed the signal statistically using Image J software.

### 2.11 Real‐Time Quantitative PCR (qPCR)

The experimental procedures were conducted according to previously described methodologies (Wang et al. 2022) with technical modifications. Briefly, total RNA was extracted from NR8383 cells with commercial kits (Bioer Technology, Hangzhou, China), followed by reverse transcription to generate complementary DNA (cDNA) (Monad Biotech, Suzhou, China). Amplification reactions were carried out with SYBR Green fluorescence dye‐containing premix employed for nucleic acid quantification (Vazyme Biotech, Nanjing, China). Detailed primer sequences were compiled in Table S2.

### 2.12 Molecular Docking

The crystal structures of the protein p65 (NF‐κB subunit, PDB ID: 4EYT), Caspase‐3 (PDB ID: 3GJQ), and KDR (VEGFR2, PDB ID: 4ASD) were obtained from the RCSB PDB database and preprocessed using PyMOL to remove water molecules and other heteroatoms. Docking between proteins was accomplished by HDOCK web server (http://hdock.phys.hust.edu.cn/).

**FIGURE 1:**
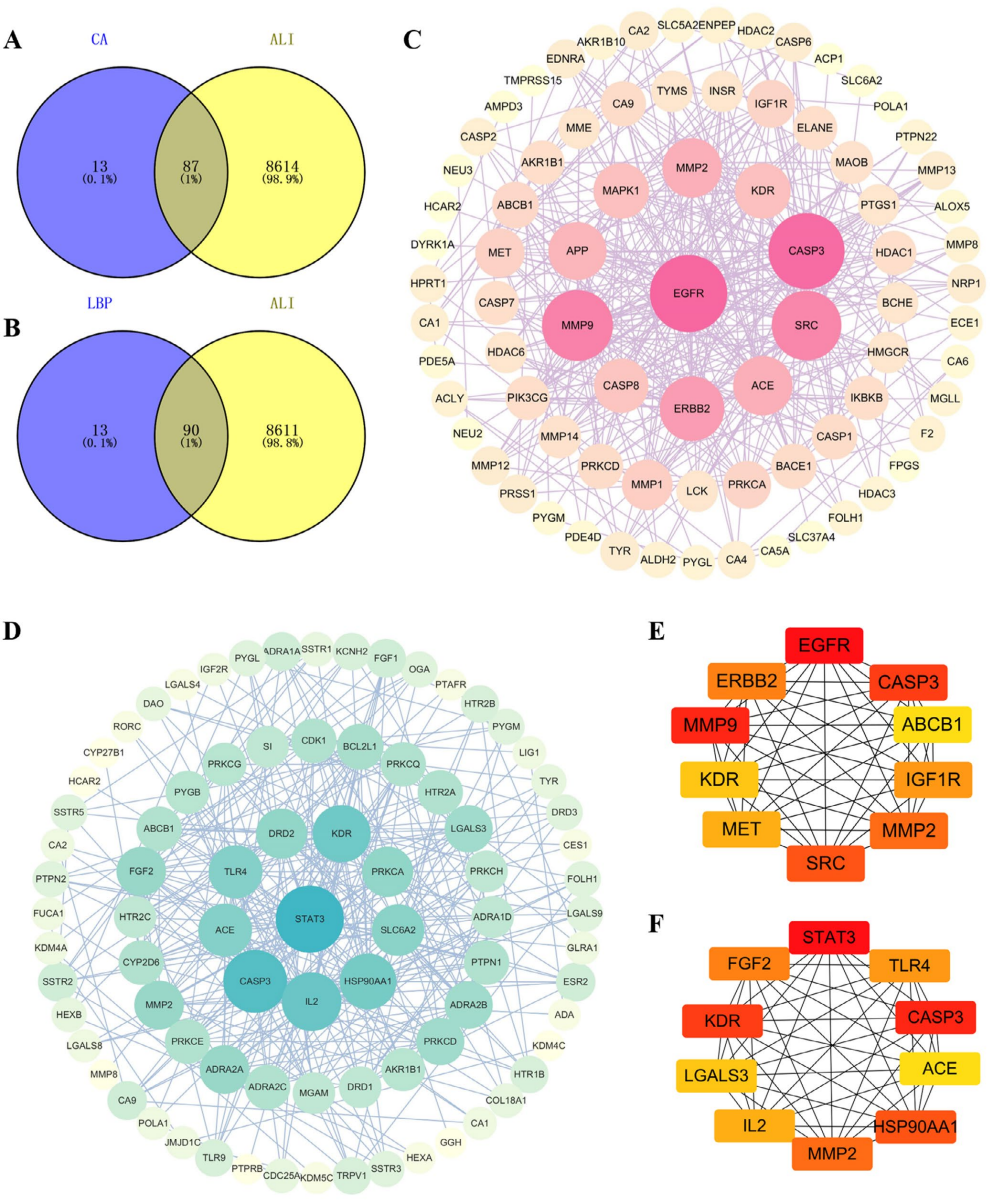
Target prediction and network construction of chlorogenic acid (CA) and *Lycium barbarum* polysaccharide (LBP) on acute lung injury (ALI). Target prediction of CA (A) and LBP (B) on ALI; Network construction of CA (C) and LBP (D) on ALI; Protein–protein interaction (PPI) network of the predicted targets of CA (E) and LBP (F) on ALI.

The ligand structures of CA and β‐1,4‐galactotriose (Figure 1) were sourced from the PubChem and ZINC databases. Open Babel was used to optimize the ligand structures and generate 3D conformations, which were then saved in PDBQT format for docking. AutoDockTools was employed to prepare the protein and ligand for docking, including charge assignment and flexible residue setup. The docking grid was centered on the protein's binding site. AutoDock performed the docking simulations using the Lamarckian genetic algorithm, with parameters optimized for computational efficiency and accuracy. PyMOL was used to visualize the protein‐ligand complexes and analyze key interactions such as hydrogen bonds and hydrophobic contacts. The binding affinities and interaction details were documented for further analysis.

### 2.13 Statistical Analysis

Analysis of variance (ANOVA) to determine the statistical significance of the data was conducted by the SPSS 22.0 software and each experiment was repeated three times. Post hoc multiple comparisons were conducted using Duncan's Multiple Range Test after ANOVA.

## 3 Result

### 3.1 Target Prediction and Network Construction of CA and LBP on ALI

A total of 100 potential targets corresponding to CA and 103 potential targets for LBP were identified. For ALI‐related targets, a comprehensive search yielded 8701 target genes. The targets associated with the compounds and those related to the disease were submitted to a Venn diagram for the identification of overlapping genes. Ultimately, 87 overlapping targets were found between CA and the disease (Figure 1A), and 90 intersecting targets were identified between LBP and the disease (Figure 1B), which were used for subsequent analysis.

Furthermore, the 87 intersecting gene targets between CA and the disease were imported into the STRING database to obtain a PPIN. This network comprised 87 nodes and 374 protein interaction edges, with an average degree value of 8.6 (Figure 1C). As shown in Figure 1D, the top 10 core targets associated with CA and ALI were epidermal growth factor receptor (EGFR), caspase 3 (CASP3), multidrug resistance protein P‐glycoprotein (ABCB1), insulin‐like growth factor 1 receptor (IGF1R), matrix metalloproteinase 2 (MMP2), tyrosine kinase C (SRC), hepatocyte growth factor receptor (MET), vascular endothelial growth factor receptor 2 (KDR), matrix metalloproteinase 9 (MMP9), and tyrosine kinase receptor 2 (ERBB2). These targets are implicated in critical processes such as epithelial repair, apoptotic cell death, and pro‐inflammatory signaling, thereby aligning with the core pathophysiology of ALI.

The PPIN of 90 intersecting genes between LBP and the disease included 89 nodes, which interacted to form 320 protein interaction edges, with an average degree value of 7.19 (Figure 1E). As shown in Figure 1F, the top 10 core targets associated with LBP and ALI were signal transducer and activator of transcription 3 (STAT3), Toll‐like receptor 4 (TLR4), CASP3, angiotensin‐converting enzyme (ACE), heat shock protein 90α family class A member 1 (HSP90AA1), MMP2, interleukin 2 (IL‐2), galectin 3 (LGALS3), KDR, and fibroblast growth factor 2 (FGF2). The functions of these targets are associated with key underlying mechanisms of the disease pathogenesis, such as hyper‐inflammatory signaling, dysregulated vascular permeability and angiogenesis, and the cellular stress response.

### 3.2 Functional and Pathway Analysis of the Alleviating Mechanism of CA and LBP on ALI

The potential biological functions of CA targets were analyzed using the Metascape database through Gene Ontology (GO) and Kyoto Encyclopedia of Genes and Genomes (KEGG) enrichment analyses. A total of 752 biological processes (BP), 68 cellular components (CC), 97 molecular functions (MF), and 130 KEGG pathways were enriched. The top 10 enriched terms based on Count values were further analyzed using the MicroSignal platform.

**FIGURE 2:**
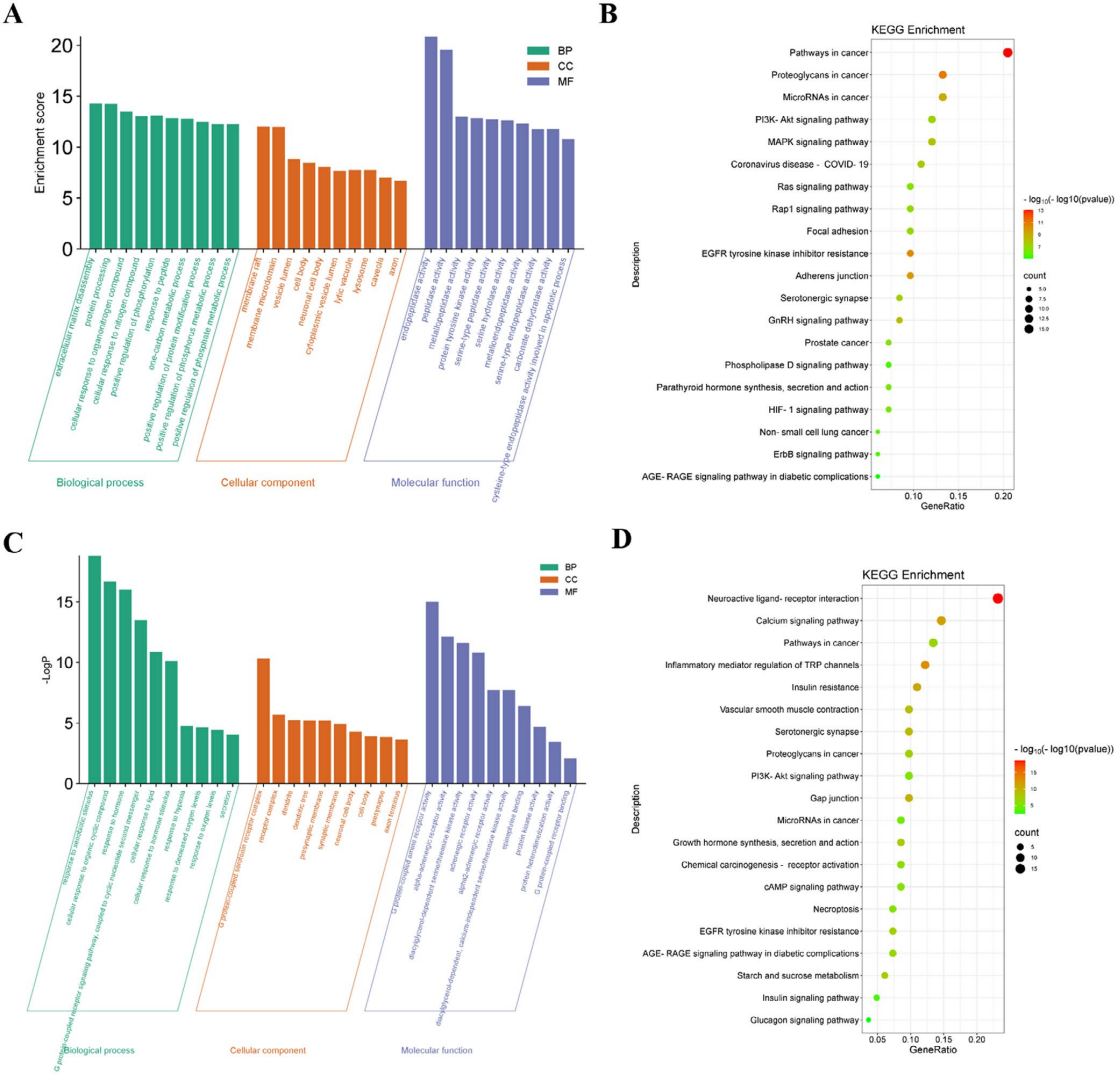
Functional and pathway analysis of the hub genes of CA and LBP on ALI. Top 30 terms of Gene Ontology (GO) functional analysis of CA (A) and LBP (C) on ALI; Top 20 pathways Kyoto Encyclopedia of Genes and Genomes (KEGG) enrichment analysis of CA (B) and LBP (D) on ALI.

As shown in Figure 2A, the enriched BP related to CA intervention in ALI included extracellular matrix degradation, protein processing, positive regulation of phosphorylation, and positive regulation of protein modification processes, which are processes that mediate cellular adaptation to ALI‐induced stress and inflammation. In terms of CC, the targets were mainly associated with membrane rafts, membrane microdomains, vesicle lumen, cell body, neuron cell body, and lysosomes. These components are essential for subcellular activities such as inflammatory factor transport and damaged protein clearance. The MF primarily involved peptidase activity, serine‐type peptidase activity, metallopeptidase activity, protein tyrosine kinase activity, and endopeptidase activity. These activities regulate proteolysis and signaling cascades central to ALI progression.

The KEGG pathway enrichment results (Figure 2B) indicated that these genes were involved in multiple pathways, including cancer pathways, corticotropin‐releasing hormone signaling pathway, MAPK signaling pathway, COVID‐19, microRNAs in cancer, PI3K‐Akt signaling pathway, lipid and atherosclerosis, IL‐17 signaling pathway, and TNF signaling pathway. Among these, the MAPK, PI3K‐Akt, IL‐17, and TNF pathways are particularly relevant to ALI, as they mediate inflammation and cell apoptosis. Notably, the enrichment of broader pathways such as “Pathways in cancer” can be explained by a significant overlap in core molecular mechanisms governing cell proliferation, apoptosis, and inflammation, which underpin the pathogenesis of ALI.

The potential biological functions of LBP targets were analyzed and a total of 801 BP, 47 CC, 90 MF, and 79 KEGG pathways were enriched. As shown in Figure 2C, the GO enrichment results indicated that the BP related to LBP intervention in ALI included cellular response to extracellular stimulus, response to hormone, adrenergic receptor signaling pathway, cellular response to nitrogen compound, and positive regulation of MAPK cascade. These processes mediate cellular adaptation to ALI‐induced stress and inflammation. In terms of CC, the targets were mainly associated with cytoplasmic vesicle lumen, vacuole lumen, lysosome, primary lysosome, and receptor complex, which are involved in inflammatory mediator degradation and signal transduction in ALI. The MF primarily involved activity of G‐protein coupled amine receptors, α‐adrenergic receptor activity, diacylglycerol‐dependent serine/threonine kinase activity, adrenergic receptor activity, and neurotransmitter receptor activity. These functions regulate ALI‐related signaling transmission.

The intersection targets of LBP and ALI were enriched into 79 pathways using the Metascape database. The top 20 KEGG pathway enrichment results indicated that these genes were involved in multiple pathways, including neuroactive ligand‐receptor interaction, cAMP signaling pathway, regulation of TRP channels by inflammatory mediators, calcium signaling pathway, starch and sucrose metabolism, growth hormone synthesis and secretion, cancer pathways, proteoglycans in cancer, PI3K‐Akt signaling pathway, and cGMP‐PKG signaling pathway (Figure 2D). Among these, the cAMP, PI3K‐Akt, and calcium pathways are critical for maintaining inflammatory homeostasis in ALI, while TRP channel regulation is linked to ALI‐induced tissue damage.

### 3.3 Multi‐Target Modulatory Effects of CA and LBP on LPS‐Induced Cells

**FIGURE 3:**
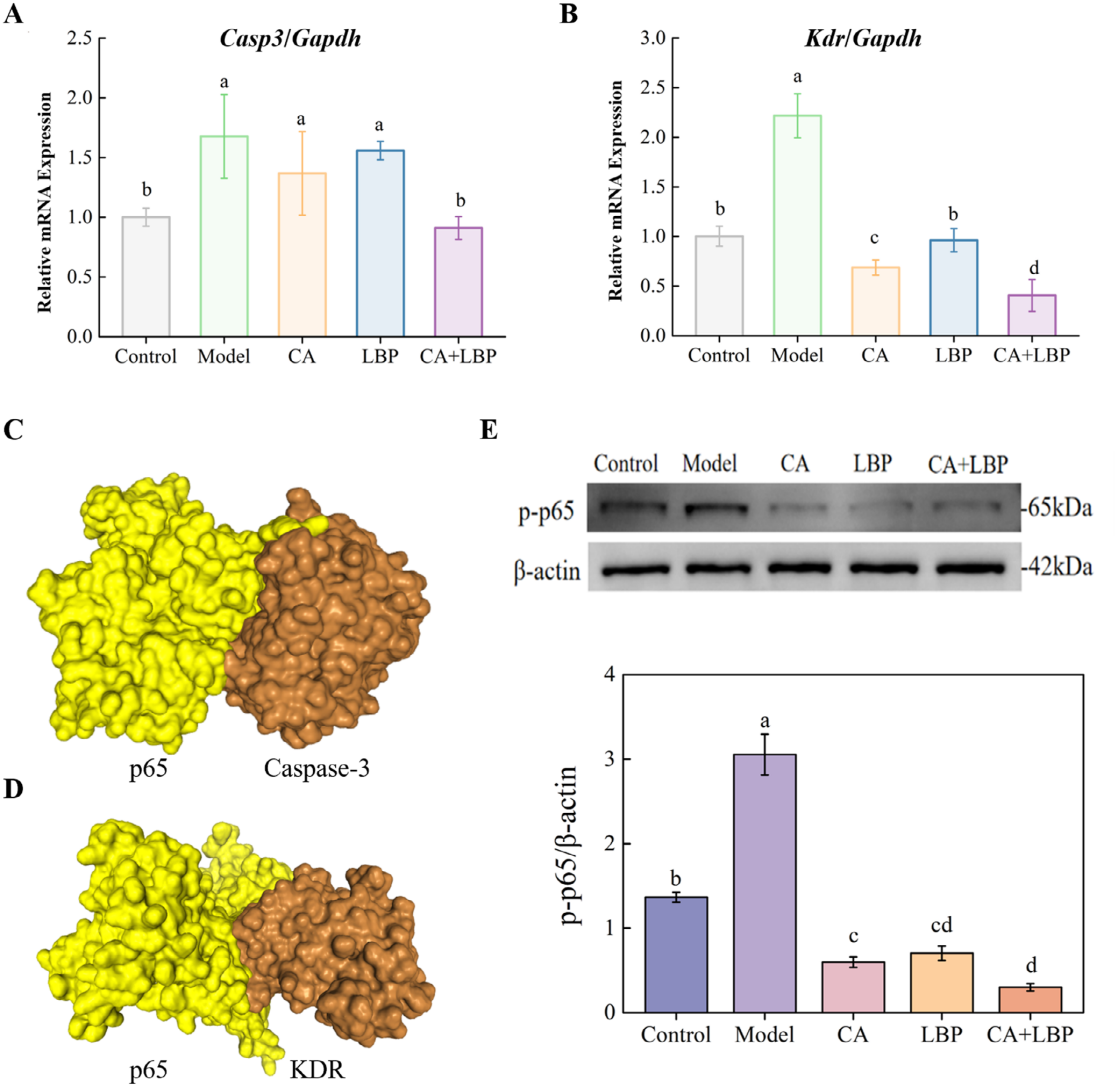
CA‐LBP alleviated lipopolysaccharide (LPS)‐induced ALI cells through multi‐targets. Relative mRNA expressions of *Casp3* (A) and *Kdr* (B), and *Gapdh* were used for normalization; Molecular docking of Casp3 (C), Kdr (D) and p65; Abundance and relative protein levels of p‐p65 (E); The results are displayed as mean ± SD (*n* = 3), dissimilar superscript letters reflect significant variations among treatments (*p* < 0.05).

To further validate the in vitro alleviating effects of CA and LBP, this study employed qPCR to examine the influence of the CA‐LBP complex on the mRNA expression levels of *Casp3* and *Kdr* in LPS‐induced NR8383 cells. Contrasted to the control, the LPS‐induced model group presented a considerable growth in *Casp3* mRNA transcription levels (Figure 3A, *p* < 0.05). While CA and LBP alone displayed no meaningful variation in *Casp3* expression compared to the model group (*p* > 0.05), the CA‐LBP complex markedly suppressed *Casp3* transcription (*p* < 0.05). Similarly, *Kdr* mRNA levels in the ALI group were markedly elevated relative to the control. Both CA, LBP, and the CA‐LBP complex inhibited LPS‐induced *Kdr* over‐expression, with the CA‐LBP combination demonstrating the most pronounced inhibitory effect (Figure 3B, *p* < 0.05).

### 3.4 Alleviating Effects of CA and LBP on NF‐κB Signaling in LPS‐Induced Cells

The combinatorial treatment of LBP and CA is highly likely to exhibit a synergistic enhancement, which is accompanied by the activation of upstream and downstream pathways on the basis of target prediction. There was a close relationship between the Casp3 (Guo et al. 2024), Kdr (Mazor et al. 2013), and NF‐κB signaling. To understand the multi‐target mechanisms by which the CA‐LBP complex alleviates LPS‐induced cellular inflammation, the protein–protein interaction network between the Casp3, Kdr, and NF‐κB was established, and the network displayed that NF‐κB interacts with both Casp3 and Kdr (Figure S2). Figure 3C,D showed that the molecular docking of Casp3 and NF‐κB, as well as Kdr and NF‐κB, exhibited robust binding affinities (Docking Score −240.38 and −248.19, respectively).

Furthermore, the abundance of key proteins in the NF‐κB signaling in NR8383 cells was analyzed. As Figure 3E exhibited, the phosphorylation level of p65 protein in the model group cells was greatly augmented compared with the control group (*p* < 0.05). Pre‐treatment with CA, LBP, and the CA‐LBP complex significantly inhibited the expression of p‐p65 in LPS‐induced macrophages, with the CA‐LBP complex exhibiting a more pronounced inhibitory effect (*p* < 0.05).

### 3.5 Alleviating Effects of CA and LBP on LPS‐Induced ALI Mice

**FIGURE 4:**
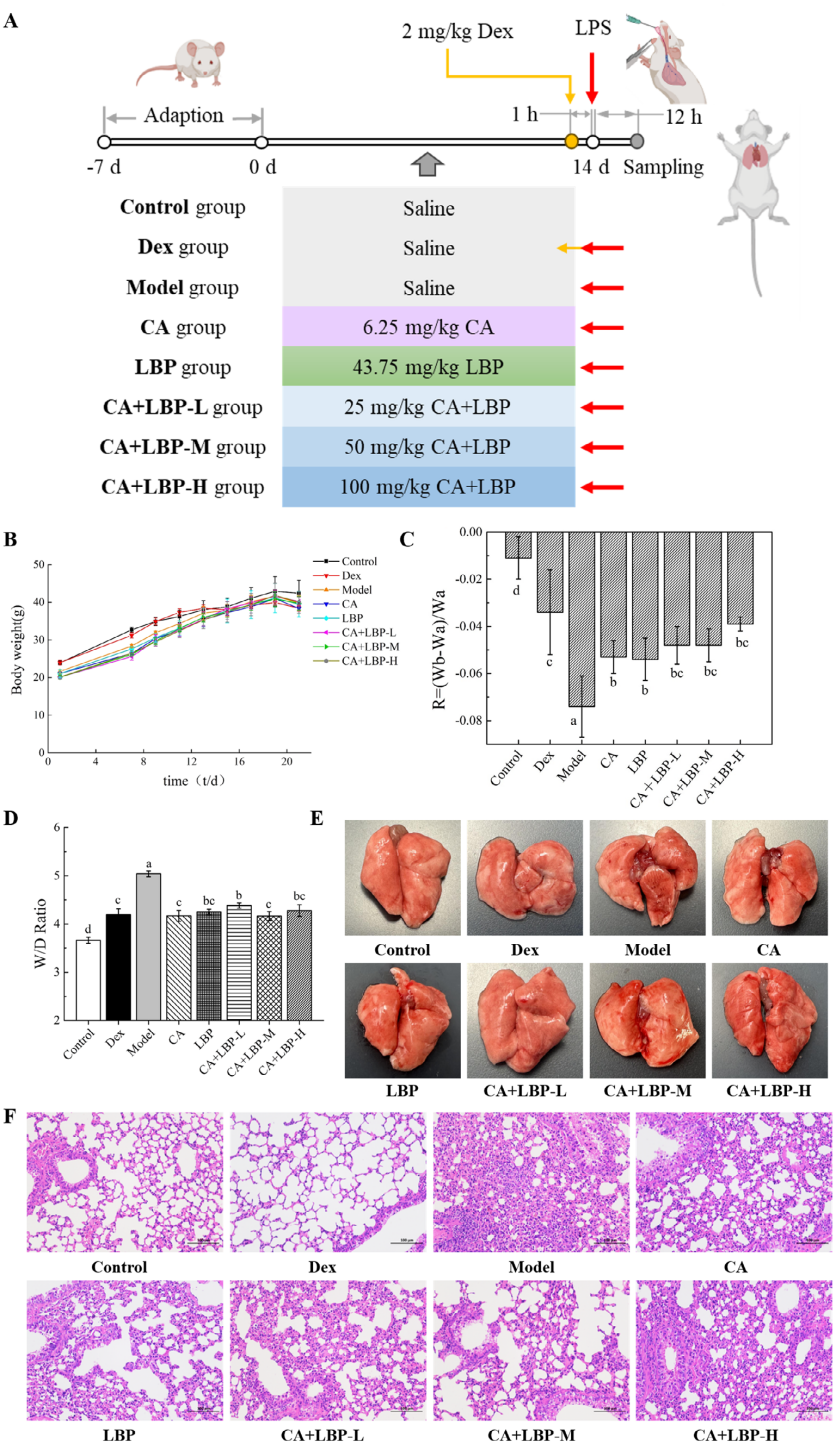
CA‐LBP alleviated LPS‐induced ALI in mice. Flow‐diagrammatic of animal handling (A); Body weight (B) and the body weight change ratio (C) of mice during the feeding period; (D) The ratio of wet lung weight to dry lung weight (W/D); The effects of CA and LBP on the morphological (E) and pathological (F) changes of lung tissue in ALI mice (HE 200×); The results are displayed as mean ± SD (*n* = 6), dissimilar superscript letters reflect significant variations among treatments (*p* < 0.05).

To validate the therapeutic potential of the CA‐LBP complex in vivo, a mouse LPS‐induced ALI model was utilized. Figure 4B showed the changes in mice body weight (BW) during the feeding period. Figure 4C illustrated the effects of CA and LBP on the BW change ratio in ALI mice. Compared with the control, the BW change ratio in the ALI group was significantly reduced (*p* < 0.05). The BW change ratio in the CA, LBP, and CA‐LBP groups was reduced compared with the model group, indicating that CA and LBP provided a certain therapeutic effect against ALI mice.

As shown in Figure 4D, compared with the control group, the wet lung weight to dry lung weight (W/D) value of lung tissue in the model group was markedly raised (*p* < 0.05). Contrasted to the ALI group, the W/D values of lung tissue in the CA, LBP, CA‐LBP groups, and the Dex group were significantly reduced. Additionally, there was no marked change between the CA, CA + LBP‐M and the Dex groups (*p* > 0.05).

As shown in Figure 4E, the lung tissue in the control group exhibited a smooth and moist surface with a sponge‐like appearance, light red in color, and was elastic without congestion. In contrast, the model group showed enlarged lung tissue due to edema, with a dull and dark red surface and signs of congestion, indicating successful induction of macroscopic lung injury. Similarly, the LBP and CA + LBP‐M/H dose groups exhibited pulmonary edema with a dark red color and localized congestion. In comparison, the Dex, CA, and CA + LBP‐L dose groups showed some relief in tissue dullness, with a pinkish hue, suggesting an ameliorative effect on LPS‐induced pulmonary edema and congestion.

The control group exhibited normal lung tissue with clear alveolar spaces, no bleeding, and no inflammatory cell infiltration (Figure 4F). Contrasted to the control, the model group showed increased infiltration of lymphocytes and granulocytes in the alveolar walls after intratracheal instillation of LPS. There was also mild thickening of large areas of the alveolar walls, widening of the alveolar septa, and the presence of macrophages and eosinophilic substances in the lumens, indicating successful modeling. Comparison with the ALI group, the CA and LBP treatment groups presented more granulocyte infiltration in the alveolar walls, with mild thickening of some alveolar walls and widening of the alveolar septa. Lung tissues of CA + LBP‐L/M/H groups showed varying degrees of improvement, with localized mild thickening of the alveolar walls and reduced inflammatory cell infiltration. These findings demonstrate that the CA‐LBP combination provides a protective effect against LPS‐induced ALI by mitigating histopathological damage.

### 3.6 Alleviating Effects of CA and LBP on Inflammation in LPS‐Induced ALI Mice

The effects of CA and LBP on pulmonary inflammation in mice were investigated by measuring the secretion levels of inflammatory cytokines induced by LPS. Compared with the control group, the expression of TNF‐α, IL‐6, and IL‐1β in the serum of mice in the model group was significantly increased (*p* < 0.05). Additionally, compared with the model group, the single components CA and LBP, as well as the CA + LBP‐L/M/H groups and the Dex group, all significantly inhibited the secretion of TNF‐α, IL‐6, and IL‐1β (*p* < 0.05).

### 3.7 Alleviating Effects of CA and LBP on Lung Injury in LPS‐Induced ALI Mice

**FIGURE 5:**
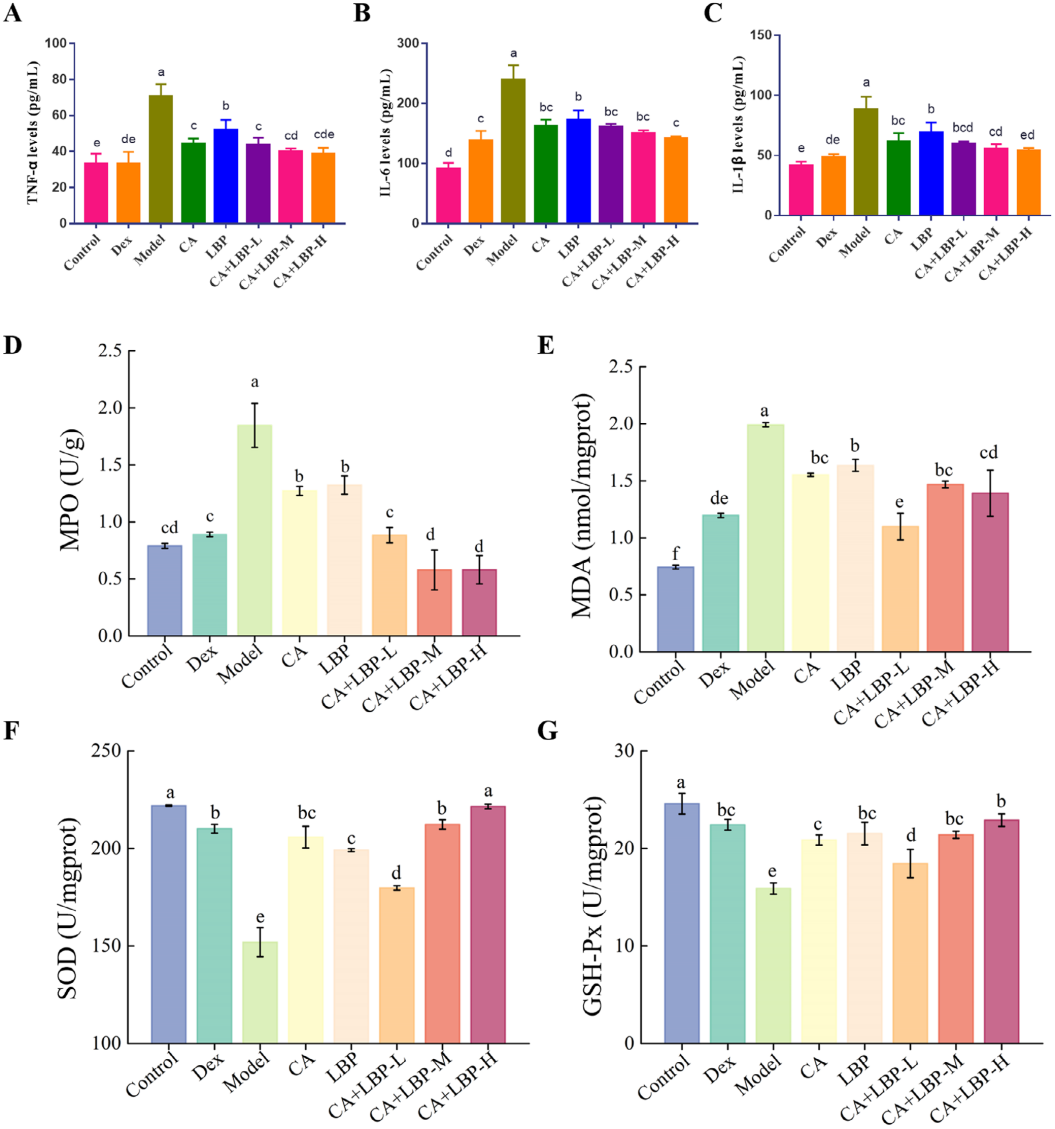
CA and LBP reversed the inflammation and lung injury index in LPS‐induced ALI mice. Levels of TNF‐α (A), IL‐6 (B), IL‐1β (C), MPO (D), MDA (E), SOD (F), and GSH‐Px (G). The results are displayed as mean ± SD (*n* = 6), dissimilar superscript letters reflect significant variations among treatments (*p* < 0.05).

As Figure 5D–G displayed, the activity of MPO and the accumulation of MDA in the lung of mice in the ALI group were significantly augmented in contrast to the control (*p* < 0.05). Meanwhile, SOD and GSH‐Px activities in the model group were markedly decreased in comparison with control (*p* < 0.05), indicating weakened antioxidant capacity. Contrast to the LPS‐induced model group, activity of MPO in the CA + LBP‐M/H groups was markedly reduced, and SOD and GSH‐Px activities were greatly increased (*p* < 0.05), suggesting that the CA‐LBP complex has the ability to enhance antioxidant capacity, inhibit pulmonary inflammatory responses, and alleviate oxidative stress caused lung injury.

### 3.8 Alleviating Effects of CA and LBP on NF‐κB Signaling in LPS‐Induced ALI Mice

**FIGURE 6:**
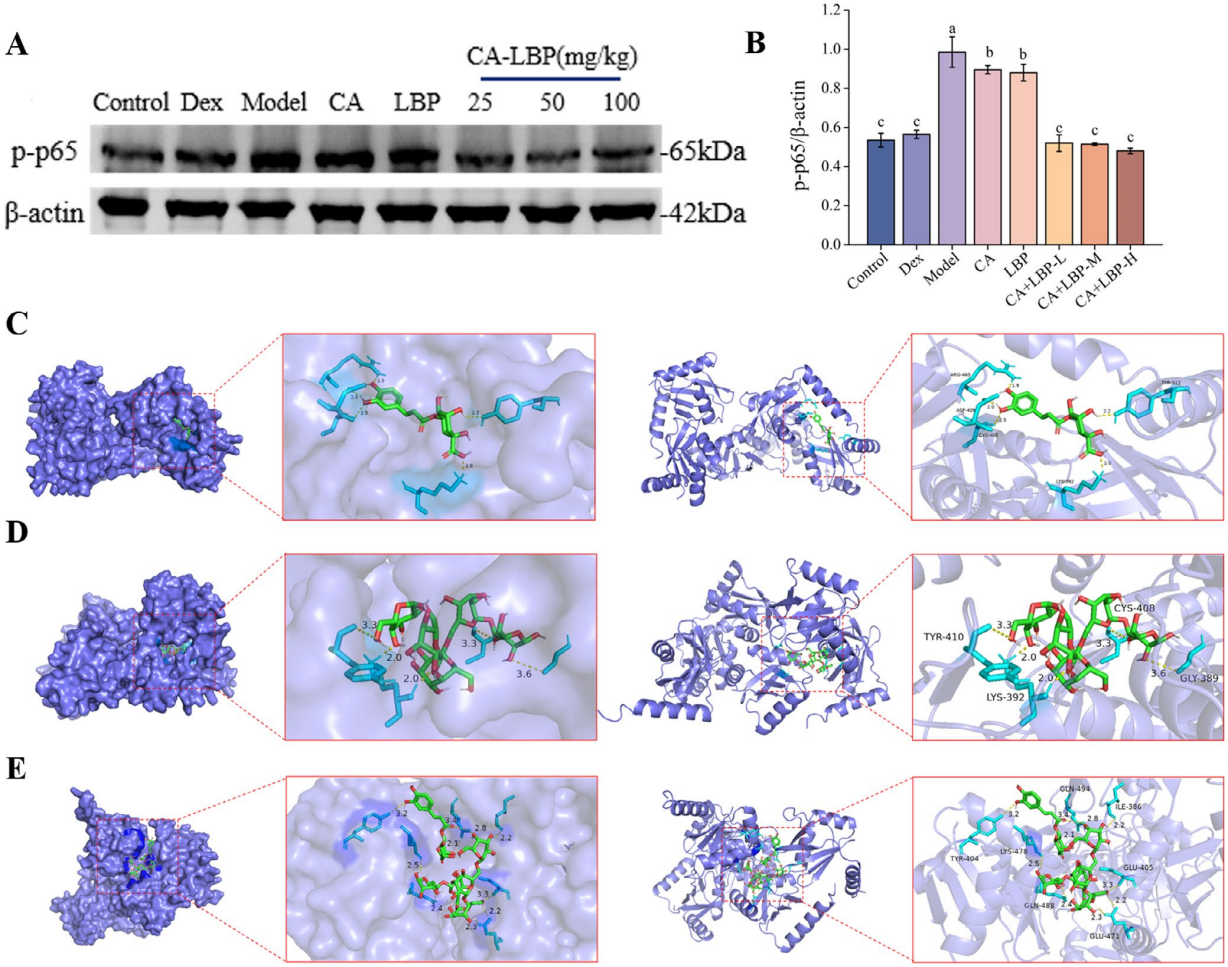
Ca and LBP suppressed NF‐κB Signaling in LPS‐induced ALI mice. Abundance (A) and relative protein levels (B) of p‐p65 and β‐Actin were used for normalization; Molecular docking of CA (C), β‐1,4‐galactotriose (D), CA‐β‐1,4‐galactotriose (E), and p65; The results are displayed as mean ± SD (*n* = 3), dissimilar superscript letters reflect significant variations among treatments (*p* < 0.05).

The expression levels of p‐p65 proteins were notably augmented in the tissues of the model group in contrast to the control (Figure 6A,B). In comparison to the ALI group, the CA + LBP‐L and CA + LBP‐H groups significantly inhibited the protein expression of p‐p65 (*p* < 0.05).

In addition, molecular docking was used to explore the ligand‐protein interactions of CA, LBP, and p65, and the interactions between CA/LBP/CA‐LBP and p65 binding site were analyzed. Our previous study showed that the main monosaccharide composed of LBP is galactose (Yin et al. 2024). Therefore, an oligosaccharide β‐1,4‐galactotriose formed by the connection of three β‐D‐galactosyl groups through β‐1,4 glycosidic bonds was constructed for exploration. There was a stable binding mode of CA/β‐1,4‐galactotriose/CA‐β‐1,4‐galactotriose and p65 complex (Figure 6C–E). Functionally, these stable binding interactions may contribute to the inhibitory effect of CA and LBP on p65 phosphorylation observed in the in vitro tissue experiments. Specifically, the interaction between CA/β‐1,4‐galactotriose/CA‐β‐1,4‐galactotriose and p65 binding site residues was analyzed using Pymol, indicating that CA was bound to the amino acid residues of p65 (CYS‐408, TYR‐513, LYS‐392, ASP‐409, and ARG‐465), β‐1,4‐galactotriose was bound to the amino acid residues of p65 (GLY‐389, TYR‐410, CYS‐408, and LYS‐392), and CA‐β‐1,4‐galactotriose was bound to the amino acid residues of p65 (GLN‐494, GLU‐405, TYR‐404, LYS‐478, GLN‐488, GLU‐471, and ILE‐386). These residue‐specific bindings may interfere with the activation of the NF‐κB pathway by disrupting the structural conformation of p65 or its interaction with upstream kinases, thereby inhibiting p‐p65 expression.

## 4 Discussion

ALI, characterized by complex pathogenesis and limited therapeutic options, remains a critical clinical challenge, necessitating innovative multi‐target strategies (Mokrá 2020). CA and LBP, bioactive natural products with anti‐inflammatory, anti‐apoptotic, and angiogenic regulatory properties, offer promising therapeutic potential for ALI. This study implemented network pharmacology to reveal the underlying mechanisms. Key targets of LBP include STAT3, CASP3, IL‐2, KDR, HSP90AA1, and MMP2, which are involved in cell proliferation, survival, and angiogenesis (Manoharan et al. 2024). In detail, STAT3, activated by cytokines such as IL‐6 and IL‐10 alongside growth factors including epidermal and fibroblast, mediates JAK/STAT signaling, a pathway critical for tumor progression and chemoresistance. CA targets include EGFR, CASP3, MMP9, SRC, KDR, and MMP2. EGFR modulates key signaling cascades like PI3K‐Akt and STAT pathways, which have been implicated in lung carcinogenesis (Gekle et al. 2023). These suggest that LBP and CA exert therapeutic effects on ALI via multi‐target, multi‐pathway interactions, providing a robust theoretical basis for their clinical translation.

Interestingly, there were three targets (CASP3, KDR, and MMP2) shared by CA and LBP. CASP3, a central executor of apoptosis, drives inflammatory gene transcription through its involvement in TNF and PI3K‐Akt pathways (J. X. Li, Han, et al. 2023). KDR, as the primary receptor for vascular endothelial growth factor (VEGF), orchestrates VEGF‐intervened processes such as cell migration, proliferation, and angiogenesis, while also contributing to airway epithelial regeneration during lung disease (Jiang et al. 2021). In this study, the CA‐LBP combination significantly suppressed the transcriptional level of *Casp3* and attenuated over‐expression of *Kdr* in LPS‐induced NR8383 cells, which further revealed the potential synergistic actions of LBP and CA against ALI through interconnected multi‐target networks.

NF‐κB, a central regulator of inflammation in ALI, promotes inflammatory gene expression upon its activation, which involves p65 phosphorylation and nuclear translocation, making it a significant pharmacological target (Cheng et al. 2024). Additionally, our previous study has shown that the CA‐LBP complex can reduce the levels of pro‐inflammatory cytokines TNF‐α and IL‐6 in LPS‐induced NR8383 cells (Yin et al. 2024), a finding that prompted us to explore the NF‐κB signaling, a master regulator controlling inflammatory response. In this study, western blot analysis revealed that the CA‐LBP complex significantly inhibited LPS‐induced p‐p65 expression in NR8383 cells. Our results indicate that the CA‐LBP complex effectively suppressed NF‐κB activation and downregulated *Casp3* transcription. These findings are consistent with the findings of Guo et al., who reported that NF‐κB directly regulates CASP3 transcription under inflammatory conditions, thereby enhancing apoptotic signaling (Guo et al. 2024). Additionally, we observed that the CA‐LBP complex modulated *Kdr* expression, which is known to be partially mediated by NF‐κB‐dependent pathways in endothelial cells during pathological angiogenesis, as described by Mazor et al. (2013). This finding suggests that CA‐LBP may exert anti‐inflammatory effects by mitigating NF‐κB activation, thereby inhibiting pro‐inflammatory cytokine expression and blocking NF‐κB‐driven upregulation of CASP3 and KDR.

It is generally acknowledged that ALI has been widely studied using LPS‐induced models (Xu et al. 2024), characterized by excessive inflammation and oxidative stress, which trigger cytokine storms and redox imbalance (Dhlamini et al. 2022). Wu et al. observed that STING inhibitor C‐176 could alleviate lung tissue injury, reduce the levels of pro‐inflammatory cytokines like TNF‐α, IL‐1β, and IL‐6, decrease the production of MPO, and alleviate oxidative injury in ALI mice induced by LPS (Wu, Xu, et al. 2022). Additionally, Zhu et al. demonstrated that natural antioxidants and anti‐inflammatory agents can mitigate ALI by suppressing NF‐κB signaling and restoring oxidative homeostasis (Zhu et al. 2023). Consistent with these findings, CA‐LBP pretreatment significantly reduced lung tissue damage, suppressed levels of pro‐inflammatory cytokines TNF‐α, IL‐1β, and IL‐6, enhanced activities of antioxidant enzymes SOD and GSH‐PX, and lowered neutrophil marker MPO and lipid peroxidation product MDA in lung tissues. Consistently, CA‐LBP suppressed p65 phosphorylation in lung tissues markedly, reinforcing that the anti‐inflammatory effects of CA‐LBP were linked to the suppression of NF‐κB activation.

In recent years, molecular docking has been broadly employed in the investigation of mechanisms (Liu et al. 2025). Tong et al. elucidated that adenanthin forms a covalent adduct with the Cys38 site on the p65, sterically hindering its DNA‐binding domain and consequently abrogating NF‐κB transcriptional activation (Tong et al. 2024). In this study, CA, β‐1,4‐galactotriose and CA‐β‐1,4‐galactotriose exhibited strong binding affinities to p65, with the CA‐β‐1,4‐galactotriose complex showing superior occupancy at key functional sites compared to β‐1,4‐galactotriose or CA alone. These computational insights suggested that CA‐LBP competitively inhibits p65 activation by blocking its phosphorylation‐related domains, consistent with our experimental observation of reduced p‐p65 expression in CA‐LBP‐treated cells and mice. The synergistic effect of CA and LBP in the complex likely enhances this inhibitory capacity, offering a structural rationale for its efficacy. These findings further validate that the CA‐LBP complex alleviates LPS‐induced ALI in mice by affecting the NF‐κB signaling pathway.

## 5 Conclusion

The present study systematically demonstrates that the CA‐LBP complex exerts a significant alleviating effect on ALI. Through in vitro experiments, it was confirmed that the CA‐LBP complex targets key genes such as *Casp3* and *Kdr*, regulates relevant pathways, and thereby inhibits the inflammatory response associated with ALI. The in vivo mouse model experiments further validated these findings. Importantly, the complex mitigates NF‐κB signaling pathway activation, with molecular docking data further corroborating this suppressive mechanism as a key mediator of its therapeutic effects.

Overall, this research not only confirms the multi‐target regulatory mechanism of the CA‐LBP complex in alleviating ALI but also provides a preliminary experimental foundation to support the exploration of novel therapeutic strategies for ALI. The findings highlight the potential of natural compound combinations like CA and LBP for the development of ALI therapeutic approaches, offering new insights into the treatment of this life‐threatening condition. Notably, this study has certain limitations. The reliance on mouse models may restrict direct translatability to human contexts, and data on the complex's toxicity or long‐term safety remain unavailable. Future research will focus on the experimental validation of the docking predictions, utilizing human primary cells and clinically relevant models to strengthen the translational relevance of our findings.

## Author Contributions

**Dandan Zhao:** project administration, resources, funding acquisition, conceptualization. **Ziyue Wang:** writing original draft, methodology. **Jianxiong Hao:** project administration, resources. **Huan Rao, Xia Zhao, Lulu Yu:** supervision, validation.

## Ethics Statement

All in vivo experiments involving mice in this study were reviewed and approved by the Ethics Committee of the Academic Committee of Hebei University of Science and Technology (Approval No. 2311095). All procedures were conducted in strict accordance with the approved protocols and complied with international guidelines for the care and use of laboratory animals.

## Conflicts of Interest

The authors declare no conflicts of interest.

## Supporting information


**Supporting Information 1:** Structure of β‐1,4‐galactotriose; Primer sequences designed for RT‐qPCR; Information about molecular docking.
**Figure S1:** Structure of β‐1,4‐galactotriose.
**Figure S2:** Protein–protein interaction network between the Casp3, Kdr and NF‐κB.
**Table S1:** Antibodies used for Western Blotting.
**Table S2:** Sequences of primers used for quantitative RT‐qPCR.
**Table S3:** Information about molecular docking.

## Data Availability

The datasets generated and analyzed during the current study are available from the corresponding author upon reasonable request.
