# Supplementary material for: Lycium barbarum Polysaccharide and Chlorogenic Acid Ameliorate LPS‐Induced Acute Lung Injury via the NF‐κB Signaling Mediated Multi‐Targets
Source: Food Sci Nutr. 2026 Jan 7;14(1):e71438. doi: 10.1002/fsn3.71438 (PMC12778399; doi:10.1002/fsn3.71438)
Supplement: Supplementary file 1 — Supporting Information 1: Structure of β‐1,4‐galactotriose; Primer sequences designed for RT‐qPCR; Information about molecular docking. Figure S1: Structure of β‐1,4‐galactotriose. Figure S2: Protein–protein interaction network between the Casp3, Kdr and NF‐κB. Table S1: Antibodies used for Western Blotting. Table S2: Sequences of primers used for quantitative RT‐qPCR. Table S3: Information about molecular docking. [file FSN3-14-e71438-s001.docx]

**Figure Legends**

Figure S1: Structure of β-1,4-galactotriose.

Figure S2: Protein-protein interaction network between the Casp3, Kdr and NF-κB.

**Figure S1**

**Figure S2**

**
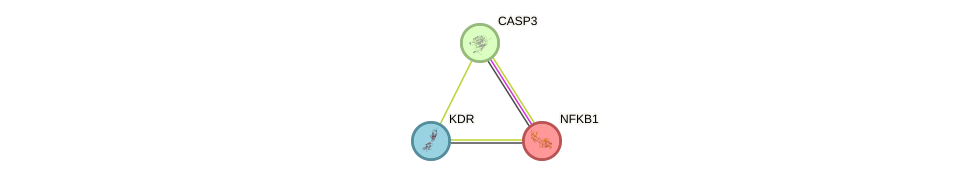
**

**Table S1 Antibodies used for Western Blotting**

| Antibody Name | Dilution Ratio | Catalog number | Antibody sources |
| --- | --- | --- | --- |
| β-actin | 1:1000 | 66009-1-Ig | Mouse |
| p-p65 | 1:1000 | 82335-1-RR | Rabbit |

*Notes: β-actin and p65 were obtained from Proteintech Group (Rosemont, USA).*

**Table S2 Sequences of primers used for quantitative RT-qPCR.**

| Gene Name | Primer (5’ - 3’) |
| --- | --- |
| *Gapdh* | F: CTGGAGAAACCTGCCAAGTATG  R: GGTGGAAGAATGGGAGTTGCT |
| *Casp3* | F: CTGGACTGCGGTATTGAGACA  R: CGGGTGCGGTAGAGTAAGC |
| *Kdr* | F: CAAGTCCGAATCCCTGTGAAGT  R: GGTGAGGATGACCGTGTAGTTTC |

**Table S3** **Information about molecular docking**

| Ligend | Protein | Final Intermolecular Energy |
| --- | --- | --- |
| CA | p65 | -7.18 kcal/mol |
| β-1,4-galactotriose | p65 | -6.71 kcal/mol |
| CA and β-1,4-galactotriose | p65 | -11.04 kcal/mol |
